# Effects of emotional valence on sense of agency require a predictive model

**DOI:** 10.1038/s41598-017-08803-3

**Published:** 2017-08-18

**Authors:** Michiko Yoshie, Patrick Haggard

**Affiliations:** 1 Human Informatics Research Institute, National Institute of Advanced Industrial Science and Technology (AIST), Tsukuba, Ibaraki, 305-8566 Japan; 2 Automotive Human Factors Research Center, AIST, Tsukuba, Ibaraki, 305-8566 Japan; 3 Institute of Cognitive Neuroscience, University College London, London, WC1N 3AR United Kingdom

## Abstract

Sense of agency (SoA), a feeling that one’s voluntary actions produce events in the external world, is a key factor behind every goal-directed human behaviour. Recent studies have demonstrated that SoA is reduced when one’s voluntary action causes negative outcomes, compared to when it causes positive outcomes. It is yet unclear whether this emotional modulation of SoA is caused by predicting the outcome valence (*prediction* hypothesis) or by retrospectively interpreting the outcome (*postdiction* hypothesis). To address this, we emulated a social situation where one’s voluntary action was followed by either another’s negative emotional vocalisation or positive emotional vocalisation. Crucially, the relation between an action and the emotional valence of its outcome was predictable in some blocks of trials, but unpredictable in other blocks. Quantitative, implicit measures of SoA based on the intentional binding effect supported the prediction hypothesis. Our findings imply that the social-emotional modulation of SoA is based on predicting the emotional valence of action outcomes.

## Introduction

The idea that humans, and perhaps many other animals, experience sense of agency (SoA), is widely accepted. However, two very different theoretical explanations of SoA have been advanced. On one view, SoA involves a retrospective attribution that an event has been caused by one’s own action, rather than another cause. Interestingly, such inferences are strongly subject to self-serving biases, such that people attribute greater agency to themselves over positive, compared to negative outcomes^1, 2^. On another view, SoA is an experience linked to the predictions or “active inferences” formed by the voluntary motor system for goal-directed action^3^. Theories of goal-directed action might imply a special link between the brain systems for computing valance and reward, and the brain systems for predictive control of action. In particular, actions are normally selected and programmed in order to produce positive rather than negative outcomes. Recent studies actually suggest that SoA is reduced for negative action outcomes compared to positive action outcomes^4, 5^. If the human SoA is indeed embedded within the brain systems for goal-directed action, one might then expect measures of SoA to track the predicted valence of action outcomes.

To objectively quantify SoA, we used a mental chronometry method to measure the intentional binding effect^6–8^. This refers to a perceived temporal compression of the interval between a voluntary action and its outcome. Ample evidence suggests that the intentional binding effect can actually capture the degree of implicit SoA^7^. Critically, intentional binding occurs when people perform movements voluntarily, but it does not occur when movements are induced involuntarily^6, 9^. Intentional binding could also be used to assess altered SoA in patients with psychiatric disorders^10, 11^. Although the relationship between intentional binding and explicit agency judgements has not been fully established, previous findings suggest that partially dissociable mechanisms underlie these two types of measures^12^.

A total of 36 native Japanese speakers (18 male; mean age ± SD = 21.8 ± 2.3 years; right-handed; university students) participated in this experiment. Participants voluntarily made a key press while viewing a continuously rotating clock. In the *predictable* conditions, their key press always produced one of four types of negative emotional vocalisations (*predictable negative* condition) or one of four types of positive emotional vocalisations (*predictable positive* condition) after a fixed delay of 250 ms. Although participants were notified whether their actions will cause negative outcomes or positive outcomes prior to each condition, the precise identity of the action outcome could not be predicted on each trial, since any of four different vocalisations with the relevant valence could be presented. In the *unpredictable* condition, their key press produced a negative vocalisation in 50% of trials and a positive vocalisation in the other 50% of trials. Importantly, in both predictable and unpredictable conditions, participants performed an identical, predetermined action (i.e., pressing a designated key rather than choosing between multiple keys) at a time of their choosing to produce different outcomes. Decisions regarding when to act have been identified as one of three classes of volitional decision (i.e., which action to execute, when to execute an action, whether to execute an action or not) that contribute to the neural basis of volition^13, 14^. The present task focused on the timing or “when” component of intentional action. This feature of the task allowed us to investigate the effects of predicting outcome valence independent of the processes of action selection.

**Figure 1:**
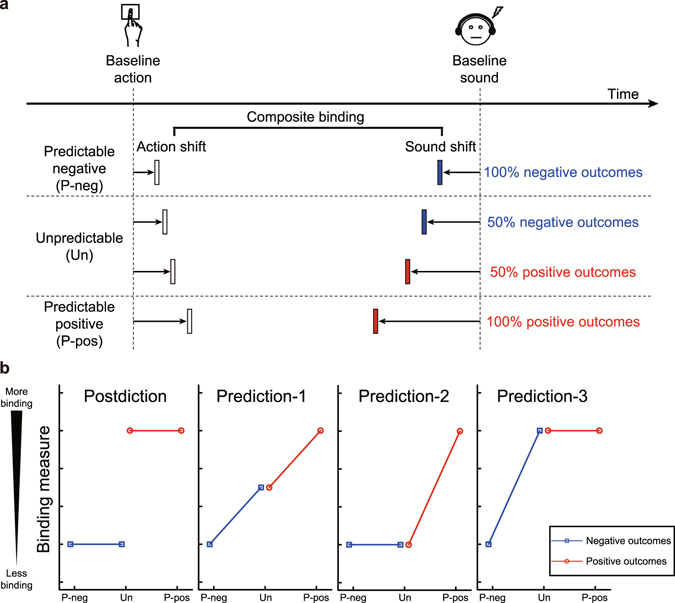
Experimental paradigm and hypotheses. (**a**) Intentional binding paradigm and the three emotional conditions. In each condition, the mean judgement error (i.e., difference between the judged and actual time of a corresponding event) in agency conditions was compared with that in single-event baseline conditions to calculate the action shift and the sound shift. We also calculated a measure of composite binding by combining the two. (**b**) Hypothetical results if the emotional modulation of SoA is postdictive (leftmost panel), and is predictive (other three panels). P-neg = predictable negative condition; Un = unpredictable condition; P-pos = predictable positive condition.

In every trial, participants were asked to report where the clock hand was at the onset of their key press or, in separate blocks, at the onset of the vocalisation. We first computed a judgement error (i.e., difference between the judged and actual time of a corresponding event) for each trial, and then compared mean judgement errors in the *agency* conditions, where the action triggered the outcome, with those in single-event *baseline* conditions (i.e., control blocks where participants pressed the key without producing the sound, or heard the sound at random intervals without pressing the key). These comparisons provided measures of how much the perceived time of the action shifted towards the sound (*action shift*), and how much the perceived time of the sound shifted towards the action (*sound shift*). Finally, we also computed a single *composite binding* measure by adding the action shift and the (sign-reversed) sound shift (Fig. 1a).

Figure 1b shows the results that would be predicted according to key theoretical hypotheses regarding the computations in the human brain that produce SoA. First, decreased SoA for predictable negative outcomes compared to predictable positive outcomes was expected from previous results^5^. More importantly, if the emotional modulation of SoA is completely postdictive (*postdiction* ^15^ hypothesis), there should be no effect of valence predictability. Binding measures would thus show no difference between trials in the predictable negative condition and trials with negative outcomes in the unpredictable condition, or between trials in the predictable positive condition and trials with positive outcomes in the unpredictable condition (Fig. 1b, leftmost panel). Conversely, if the emotional modulation of SoA is predictive (*prediction* hypothesis), differences in binding measures between negative outcome trials and positive outcome trials should occur in the predictable condition, but not in the unpredictable condition. In this case, there are three possible patterns of hypothetical results: (1) The predictability of outcome valence affects binding measures equivalently for negative and positive outcomes (Fig. 1b, second panel from the left), (2) The predictability of outcome valence affects binding measures more strongly for positive outcomes than for negative outcomes (Fig. 1b, third panel from the left), and (3) The predictability of outcome valence affects binding measures more strongly for negative outcomes than for positive outcomes (Fig. 1b, rightmost panel). Here we sought to clarify which of these hypotheses can best explain the emotional modulation of SoA.

## Results and Discussion

**Figure 2:**
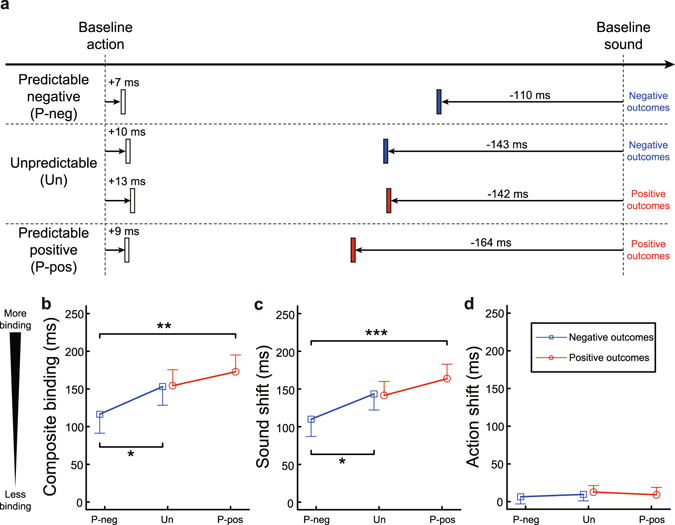
Measures of intentional binding in each emotional condition. (**a**) Summary of the results. (**b**) Means of the composite binding. (**c**) Means of the sound shift (sign-reversed for illustration purposes). (**d**) Means of the action shift. Data are represented as mean ± SEM. P-neg = predictable negative condition; Un = unpredictable condition; P-pos = predictable positive condition. *P < 0.05; **P < 0.01, ***P < 0.001.

Figure 2a provides the summary of the results. We first performed a two-way ANOVA on the composite binding, with valence predictability (predictable vs. unpredictable) and outcome valence (negative vs. positive) as factors (Supplementary Table S1; Fig. 2b). It revealed a significant main effect of outcome valence (*F*(1, 35) = 7.14, *P* = 0.011, partial η^2^ = 0.17), as well as a significant two-way interaction (*F*(1, 35) = 8.50, *P* = 0.006, partial η^2^ = 0.20). To explore this interaction, we subsequently performed simple effects tests. We first compared the two types of outcome valence in each condition of valence predictability. In the predictable condition, the composite binding was significantly smaller for the negative than positive outcomes (*t*(35) = −3.81, *P* = 0.001, *d* = 0.40, mean difference = −56.33, 95% CI of the difference = [−86.38, −26.28], paired *t*-test, two-tailed). The result confirmed that the intentional binding effect becomes smaller for predictable negative outcomes than for predictable positive outcomes, replicating a previous finding^5^. We next compared the two types of outcome valence in the unpredictable condition. If the emotional modulation of SoA includes a postdictive component, there should be a significant difference between trials with negative outcomes and those with positive outcomes. The composite binding demonstrated no significant difference between the negative and positive outcomes (*t*(35) = −0.09, *P* = 0.931, *d* = 0.05, mean difference = −1.21, 95% CI of the difference = [−29.30, 26.87], paired *t*-test, two-tailed), providing no evidence for a postdictive contribution to the emotional modulation of SoA. We then tested for simple effects of valence predictability for each type of outcome valence. We found a significant effect of valence predictability for negative outcomes. That is, the composite binding for negative outcomes was significantly smaller in the predictable than in the unpredictable condition (*t*(35) = −2.23, *P* = 0.032, *d* = 0.25, mean difference = −36.61, 95% CI of the difference = [−69.94, −3.28], paired *t*-test, two-tailed). As for the positive outcomes, on the other hand, there was no significant difference in the composite binding between the predictable and unpredictable conditions (*t*(35) = 1.29, *P* = 0.204, *d* = 0.14, mean difference = 18.51, 95% CI of the difference = [−10.52, 47.54], paired *t*-test, two-tailed). The predictions about outcome valence thus modulated SoA when participants’ voluntary action triggered negative outcomes, but not when their action triggered positive outcomes.

The binding effect can be decomposed into a shift in the perceived time of actions forward towards their outcomes (*action shift*), and an additional shift in the perceived time of outcomes back towards the actions that caused them (*sound shift*). We therefore analysed each of these shifts separately, using the same design as before (see Supplementary Data). We first performed a two-way ANOVA of valence predictability × outcome valence on the sound shift (Supplementary Table S2; Fig. 2c). It revealed a significant main effect of outcome valence (*F*(1, 35) = 7.09, *P* = 0.012, partial η^2^ = 0.17), along with a significant two-way interaction (*F*(1, 35) = 10.32, *P* = 0.003, partial η^2^ = 0.23). We thus tested for simple effects of outcome valence in each condition of valence predictability. Although the perceived time of the sounds shifted back towards the actions less strongly for the negative than positive outcomes in the predictable condition (*t*(35) = 4.16, *P* < 0.001, *d* = 0.43, mean difference = 53.74, 95% CI of the difference = [27.51, 79.96], paired *t*-test, two-tailed), the same contrast was insignificant in the unpredictable condition (*t*(35) = −0.15, *P* = 0.881, *d* = 0.02, mean difference = −1.98, 95% CI of the difference = [−28.64, 24.68], paired *t*-test, two-tailed). We then tested for simple effects of valence predictability for each type of outcome valence. For negative outcomes, the perceived time of the sounds shifted back towards the actions less strongly in the predictable than unpredictable condition (*t*(35) = 2.22, *P* = 0.033, *d* = 0.25, mean difference = 33.52, 95% CI of the difference = [2.91, 64.13], paired *t*-test, two-tailed). For positive outcomes, only a marginally significant trend was found between the predictable and unpredictable conditions (*t*(35) = −1.71, *P* = 0.096, *d* = 0.20, mean difference = −22.20, 95% CI of the difference = [−48.56, 4.17], paired *t*-test, two-tailed). The results of the sound shift were generally consistent with those of the composite binding.

Finally, we conducted the same analyses on the action shift. A two-way ANOVA of valence predictability × outcome valence showed that neither the main effects of the two factors nor the interaction effect were significant (Supplementary Table S3; Fig. 2d). This indicates that the effects found for the composite binding are mainly explained by the changes in the sound shift.

All in all, these results support our prediction hypothesis. The intentional binding effect was significantly reduced for predictable negative compared to predictable positive outcomes, corroborating previous findings^5^. When the emotional valence of action outcomes was unpredictable, however, the binding effect did not significantly differ between the negative and positive outcomes. This indicates that the emotional component of SoA may not involve postdiction. In contrast, predictions about outcome valence seemed to mediate the emotional effects on SoA. The binding effect for negative outcomes was weaker in the predictable than in the unpredictable condition, as shown by analyses of both composite binding and sound shift. For positive outcomes, the difference in the binding effect between the predictable and unpredictable conditions was smaller than for negative outcomes, and only a marginally significant difference was found for the sound shift. Therefore, the predictability of outcome valence seems to have affected SoA more strongly for negative outcomes than for positive outcomes, supporting the third possible pattern of results consistent with the prediction hypothesis (Fig. 1b, rightmost panel). Previous studies showed that prime-induced predictions about action outcomes enhance SoA^16, 17^. Our findings indicate that the emotional content of outcome predictions can further modulate SoA.

Importantly, our design manipulated only the predictability of outcome valence, not the predictability that the outcome would occur. Since some outcome was always present in the agency conditions, outcome occurrence was always predictable. One recent study suggested that valence effects were largely postdictive when outcome occurrence was unpredictable^18^. Thus, predicting outcome occurrence and predicting outcome valence may stand in a hierarchical relation, with the former being necessary for the latter. This suggests that one must first build a reliable representation that one’s action causes an outcome, in order for the predictive emotional modulation of SoA to occur. Importantly, the specific outcome token was never predictable in our design, since we selected at random from four different vocalisations for each condition of emotional valence. While participants could predict *which* emotional valence (i.e., negative or positive) they would experience during the following block in the predictable condition, they could never predict which precise vocalisation they would receive on each trial. These features of our design strengthen the view that SoA depends crucially on whether participants predicted the *valence* of action outcome, rather than predicting its mere occurrence, or predicting a specific token identity. In that sense, our results reflect the influence of a valence system on predictive motor cognition, which cannot readily be explained by other means.

Our experimental paradigm did not allow us to formally distinguish between facilitatory effects of positive predictions and inhibitory effects of negative predictions on binding, since we did not include a neutral outcome condition. However, a previous study showed that the binding costs of negativity were more marked than the binding benefits of positivity^5^. Consistent with this pattern, the current results indicated that the predictability of outcome valence affected SoA more substantially over negative, compared to positive outcomes. We thus argue that predicting negative outcomes of one’s voluntary action attenuates SoA.

Previous findings suggest that outcome valence modulates the intentional binding effect mainly through changes in the sound shift^4, 5^. The present results also demonstrated that neither outcome valence, nor predictability of outcome valance modulated the action shift. This suggests that the predictable valence affects SoA primarily by altering the representation of outcomes, by adjusting the strength of the linkage between outcomes and actions, rather than vice versa.

In the present task, participants repeatedly performed a single action, but produced different outcomes, enabling us to isolate the effects of predicting outcome valence. Future work should further examine how the processes of action selection interact with processes of outcome valence prediction and influence SoA. In addition, the present task was based on a situation where different emotional conditions were fully controlled, and participants acted alone. This allowed a level of controllability and precision which is useful for experimental power, but differs from everyday emotional situations. In particular, the social reciprocity and adaptive nature of many natural emotional interactions were absent from our study. Future work might adopt a more naturalistic social situation and investigate whether the present findings also apply to real-world social contexts. Finally, since the present sample was limited to Japanese university students, extending this work to individuals with a variety of cultural and educational backgrounds would be worthwhile in the future.

We conclude that a specialised cognitive mechanism reduces SoA when people perform actions that they predict will have negative outcomes. One functional role of this mechanism may be to provide a form of emotional distancing^19, 20^ when people perform actions that they know will produce unpleasant outcomes. A recent study showed that such distancing mechanisms also occur when performing actions under social coercion^21^. The present results suggest that performing actions with reliably unpleasant outcomes may reduce the experience of voluntary control, compared to performing actions with reliably more pleasant outcomes. Many views of human nature emphasise pursuit of positive goals, and the importance of agency for normal human well-being. In terms of brain mechanisms, the concept of goal-directed action implies some linkage between the brain’s valence systems and voluntary action systems. Our results imply that the experience of one’s own agency may reflect this linkage. This suggests one important way in which our experience of agency tracks the actual underlying neural computations for action, rather than being merely a retrospective confabulation.

Finally, our results have potential applications to psychopathology. We have identified a mechanism whereby routinely negative action outcomes could influence a core component of the self, namely SoA. In future research we will investigate how this link between predictable negative valence and SoA could affect well-being and psychopathology.

## Methods

### Participants

Thirty-six native Japanese speakers (18 male; mean age ± SD = 21.8 ± 2.3 years) participated in the main experiment. All participants had normal or corrected-to-normal vision, and were without auditory impairment or history of psychiatric or neurological illness. All were right handed, with a mean (±SD) Edinburgh Handedness Inventory^22^ score of 86.8 (±16.4). Participants were recruited at the University of Tsukuba, and consisted of both undergraduate and graduate students. Participants gave written informed consent prior to the experiment. The study was approved by the National Institute of Advanced Industrial Science and Technology (AIST) Ethics Committee, and was carried out in accordance with the approved guidelines.

To determine the sample size, we first performed a power calculation by using G*Power 3.1.9.2^23^. We estimated the minimum number of participants necessary to identify a significant difference between the predictable negative and predictable positive conditions (effect size = 0.618; alpha = 0.05: power = 0.90) based on a previous study^5^. This analysis indicated that a minimum of 30 participants were necessary for the main experiment. Since we needed to fully counterbalance potentially confounding order effects: order of emotional conditions (predictable negative, predictable positive, and unpredictable; _3_P_3_ = 6 patterns) and order of judged events (action first or sound first; 2 patterns), we chose to recruit a total of 36 participants (the minimum multiple of 12 above 30), corresponding to 12 patterns × 3 repetitions. One participant spontaneously reported being depressed at the end of the testing session. The data obtained from this participant were excluded prior to analyses, and a replacement participant was tested.

### Auditory stimuli

Eight nonverbal emotional vocalization stimuli were used to manipulate the emotional valence of action outcomes. We developed the stimuli for native Japanese speakers to match the English stimuli used in a previous study^5^. The Japanese stimuli were recorded in an anechoic chamber using an audio recorder H6 with a shotgun microphone (Zoom, Tokyo, Japan). Nine Japanese actors (five male; mean age ± SD = 25.9 ± 7.0 years) were presented with a brief scenario for two negative (fear and disgust) and two positive (achievement and amusement) emotions, and were asked to produce 12 vocalisations that they would be likely to make in each situation^24^. The sounds were digitized at a 44.1 kHz sampling rate and their peak amplitude was normalised.

**Figure 3:**
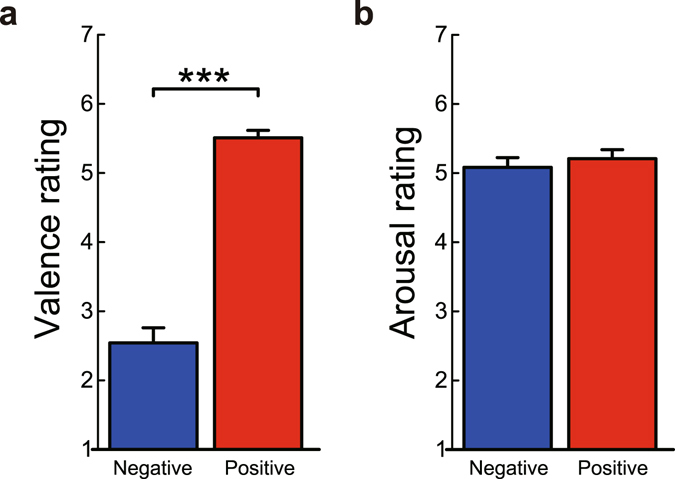
Subjective ratings of auditory stimuli. (**a**) Perceived emotional valence of auditory stimuli. Participants rated positive vocalisations to be more positive than negative vocalisations. (**b**) Perceived emotional arousal of auditory stimuli. There was no difference in arousal rating between negative and positive vocalisations, confirming that we could effectively manipulate emotional valence of action outcomes while controlling for emotional arousal. Data are represented as mean ± SEM. ***P < 0.001.

All of the recorded vocalisations were then pilot tested with 16 native Japanese speakers (eight male; mean age ± SD = 20.8 ± 1.9 years) to exclude poor exemplars. The 16 pilot participants performed a forced-choice task, and the best recognised eight male and eight female tokens were selected for each emotion. These vocalisations were then tested with another group of 30 native Japanese speakers (15 male; mean age ± SD = 21.0 ± 2.1 years) to produce the final stimulus set. The 30 participants were asked to rate 1) the perceived valence of the expressed emotion on a seven-point scale ranging from 1 (*extremely negative*) to 7 (*extremely positive*), and 2) the perceived arousal of the expressed emotion on a seven-point scale ranging from 1 (*completely calm*) to 7 (*extremely excited*). Based on these ratings, we selected four negative vocalisations (two fear and two disgust sounds of a male and a female each) and four positive vocalisations (two achievement and two amusement sounds of a male and a female each) that significantly varied in perceived valence (Fig. 3a; *t*(29) = 12.10, *P* < 0.001, *d* = 3.14, mean difference = 2.97, 95% CI of the difference = [2.47, 3.47], paired *t*-test, two-tailed), but not in perceived arousal (Fig. 3b; *t*(29) = 0.91, *P* = 0.369, *d* = 0.17, mean difference = 0.13, 95% CI of the difference = [−0.16, 0.41], paired *t*-test, two-tailed), similarly to the original English stimuli^5^. The auditory stimuli in each category were carefully matched for pitch and duration. All the stimuli were presented by headphones (Sennheiser HD380 Pro; Sennheiser, Wedemark, Germany).

### Experimental task and procedure

The intentional binding task was presented to participants using a customised program running in LabVIEW 2010 (National Instruments, Austin, TX, USA)^5, 6^. Participants viewed a clock face marked with conventional intervals (5, 10, 15, …, and 60) on a 20.1-inch flat screen (Dell E207WFPc; Dell, Round Rock, TX, USA). On each trial the clock hand (length: 12 mm) started to rotate from a random position on the clock face, at a rate of 2,560 ms per cycle. Participants then performed one of four different tasks: in *agency* conditions, they were asked to press a key on a silent silicone computer keyboard with the right index finger at a time of their choosing, which caused a sound 250 ms later. After the sound’s offset the clock hand continued rotating for a random time (1,320–2,750 ms), and then stopped. Participants were then prompted to verbally report where the clock hand was at the onset of their keypress (*agency action* condition) or, in a separate block, at the onset of the sound (*agency sound* condition). In the *baseline action* condition, participants similarly pressed a key at a time of their choosing, but this keypress did not cause a sound. After the clock hand has stopped, they judged the time of their keypress. In the *baseline sound* condition, on the other hand, participants passively heard sounds at random intervals, which mimicked time intervals of their voluntary keypress, and judged the time of each sound onset. To make sure that participants understood the task, we asked participants to perform five practice trials before moving on to a different condition using a neutral beep of 70-ms duration. In addition, before the testing session, we asked participants to practise pressing the key without making any clicking sounds in order to prevent clicking sounds from affecting the results.

We used this paradigm to investigate whether SoA is influenced by the existence of predictions about the emotional valence of action outcomes. To this end, participants were presented with three different emotional conditions: In the *predictable negative* condition, each keypress was always followed by one of four negative vocalisations, and participants could predict that their action would cause a negative outcome. In the *predictable positive* condition, on the other hand, each keypress was always followed by one of four positive vocalisations, and participants could predict that their action would cause a positive outcome. For these two *predictable* conditions, participants underwent three task blocks of 32 trials each, corresponding to the agency action, agency sound, and baseline sound conditions (32 trials × 1 block × 3 task conditions × 2 emotional conditions). In each block four different sounds with the relevant valence were presented in a randomised order (4 sounds × 8 repetitions). In the *unpredictable* condition, each keypress was followed by a negative vocalisation on 50% of trials and by a positive vocalisation on the other 50% of trials, and participants could not predict whether their action would cause a negative or positive outcome. For this condition, participants underwent two task blocks each for the agency action, agency sound, and baseline sound conditions (32 trials × 2 blocks × 3 task conditions). In each block, two sounds (one male and one female vocalisation) of a positive emotion (i.e., achievement or amusement) and two sounds of a negative emotion (i.e., fear or disgust) were presented in a randomised order: Half of participants (*N* = 18) heard achievement and fear sounds in one block, amusement and disgust sounds in another block. The other half (*N* = 18) heard achievement and disgust sounds in one block, amusement and fear sounds in another block. In both groups, the order of the two unpredictable blocks were reversed in half of participants (*N* = 9). In addition, participants performed one common baseline action block, leading to a total of 13 task blocks (32 trials × 13 blocks = 416 trials).

The whole experiment was divided into two sessions. Each session was devoted to action judgements (agency action and baseline action conditions) or sound judgements (agency sound and baseline sound conditions) only. Half of participants (*N* = 18) judged the times of actions in the first session and those of sounds in the second session, while in the other half (*N* = 18) the order was reversed. A 10-min break was inserted between the two sessions. The order of the three emotional conditions (predictable negative, predictable positive, and unpredictable; _3_P_3_ = 6 patterns) was consistent within a participant and was completely counterbalanced between participants (6 patterns × 6 repetitions) to avoid order effects. The order of the task conditions (agency first or baseline first) was randomly chosen for each participant and completely counterbalanced (18 vs. 18 participants). Within the sound judgement session, the agency and baseline task blocks of an emotional condition were presented successively, and a 5-min break was inserted before moving on to another emotional condition.

For all of the three emotional conditions, each block was further divided into two sub-blocks of 16 trials each and the repetitions of the four sounds were manipulated to be unevenly distributed across the sub-blocks (i.e., Each sound appeared twice, three, five, or six times within each sub-block. In the unpredictable condition, we ensured that the number of negative sounds equalled to that of positive sounds within each sub-block). To help participants maintain vigilance and to encourage their attention to the emotional features of the auditory stimuli, we asked participants to rank the frequency of the four sounds at the end of every sub-block. The instructions about these additional questions were given to participants just prior to each sub-block: We first let participants hear all four sounds that would be presented in the following sub-block, and told them that they would be asked to rank the frequency of these sounds at the end of the sub-block. These data are presented in Supplementary Results and Supplementary Table S4.

### Calculation of binding measures

We first computed a judgement error for each trial by subtracting the actual onset time of an action or a sound from the judged onset time of the corresponding event. A positive judgement error indicated a delayed judgement, while a negative error indicated an anticipatory judgement. Next, we averaged judgement errors across all 32 trials in the predictable negative condition, 32 trials in the predictable positive condition, 32 trials with negative outcomes in the unpredictable condition, and 32 trials with positive outcomes in the unpredictable condition.

We then computed the mean shift in the perceived time of actions by subtracting the mean judgement error in the baseline action condition from that in the agency action condition for each emotional condition (*action shift*). In the same way, we computed the mean shift in the perceived time of sounds in the agency sound condition relative to the baseline sound condition for each emotional condition (*sound shift*). Although the sound shift generally tends to be larger than the action shift^6^, this asymmetry becomes especially pronounced when emotional vocalization stimuli are used for action outcomes^5^. This is presumably because the ambiguity and long duration of vocalization stimuli lead to variant onset judgements. Finally, we also quantified the overall subjective temporal association between action and outcome by combining the action shift and the sign-reversed sound shift (*composite binding*). All behavioural data were analysed in Matlab 8.4 (MathWorks, Natick, MA, USA) using purpose-written routines.

### Statistical analyses

The three measures of intentional binding (i.e., action shift, sound shift, and composite binding) were first analysed with two-way ANOVAs of valence predictability (predictable vs. unpredictable) × outcome valence (negative vs. positive). When the two-way interaction was found to be significant, we subsequently performed simple effects tests in two ways to explore the origin of the interaction: We first compared the two types of outcome valence (negative vs. positive) in each condition of valence predictability by performing two-tailed paired *t*-tests. We similarly compared the two conditions of valence predictability (predictable vs. unpredictable) for each type of outcome valence. For all of the statistical tests of the main experiment, *N* was 36, and the *P* value < 0.05 was regarded as significant.

### Data availability

The data that support the findings of this study are included in Supplementary Information.

## Electronic supplementary material

Supplementary Information

Dataset 1

## References

[1] Bradley GW (1978). Self-serving biases in attribution process: re-examination of fact or fiction question. J. Pers. Soc. Psychol.

[2] Greenberg J, Pyszczynski T, Burling J, Tibbs K (1992). Depression, self-focused attention, and the self-serving attributional bias. Pers. Individ. Dif..

[3] Adams RA, Shipp S, Friston KJ (2013). Predictions not commands: active inference in the motor system. Brain Struct. Funct..

[4] Takahata K (2012). It’s not my fault: postdictive modulation of intentional binding by monetary gains and losses. PLoS One.

[5] Yoshie M, Haggard P (2013). Negative emotional outcomes attenuate sense of agency over voluntary actions. Curr. Biol..

[6] Haggard P, Clark S, Kalogeras J (2002). Voluntary action and conscious awareness. Nat. Neurosci..

[7] Moore JW, Obhi SS (2012). Intentional binding and the sense of agency: a review. Conscious. Cogn..

[8] Haggard P (2017). Sense of agency in the human brain. Nat. Rev. Neurosci..

[9] Haggard P, Clark S (2003). Intentional action: conscious experience and neural prediction. Conscious. Cogn..

[10] Voss M (2010). Altered awareness of action in schizophrenia: a specific deficit in predicting action consequences. Brain.

[11] Haggard P, Martin F, Taylor-Clarke M, Jeannerod M, Franck N (2003). Awareness of action in schizophrenia. Neuroreport.

[12] Ebert JP, Wegner DM (2010). Time warp: authorship shapes the perceived timing of actions and events. Conscious. Cogn..

[13] Brass M, Haggard P (2008). The what, when, whether model of intentional action. Neuroscientist.

[14] Zapparoli L, Seghezzi S, Paulesu E (2017). The what, the when, and the whether of intentional action in the brain: a meta-analytical review. Front. Hum. Neurosci..

[15] Eagleman DM, Sejnowski TJ (2000). Motion integration and postdiction in visual awareness. Science.

[16] Moore JW, Wegner DM, Haggard P (2009). Modulating the sense of agency with external cues. Conscious. Cogn..

[17] Gentsch A, Kathmann N, Schutz-Bosbach S (2012). Reliability of sensory predictions determines the experience of self-agency. Behav. Brain Res..

[18] Christensen JF, Yoshie M, Di Costa S, Haggard P (2016). Emotional valence, sense of agency and responsibility: a study using intentional binding. Conscious. Cogn..

[19] Koenigsberg HW (2010). Neural correlates of using distancing to regulate emotional responses to social situations. Neuropsychologia.

[20] Koenigsberg HW (2009). Neural correlates of the use of psychological distancing to regulate responses to negative social cues: a study of patients with borderline personality disorder. Biol. Psychiatry.

[21] Caspar EA, Christensen JF, Cleeremans A, Haggard P (2016). Coercion changes the sense of agency in the human brain. Curr. Biol..

[22] Oldfield RC (1971). Assessment and analysis of handedness: Edinburgh inventory. Neuropsychologia.

[23] Faul F, Erdfelder E, Lang A-G, Buchner A (2007). G*Power 3: A flexible statistical power analysis program for the social, behavioral, and biomedical sciences. Behav. Res. Methods.

[24] Sauter DA, Eisner F, Calder AJ, Scott SK (2010). Perceptual cues in nonverbal vocal expressions of emotion. Q. J. Exp. Psychol..

