# Supplementary material for: Effects of emotional valence on sense of agency require a predictive model
Source: Sci Rep. 2017 Aug 18;7:8733. doi: 10.1038/s41598-017-08803-3 (PMC5562802; doi:10.1038/s41598-017-08803-3)
Supplement: Supplementary file 1 — Supplementary Information [file 41598_2017_8803_MOESM1_ESM.pdf]

Supplementary Information for

Effects of emotional valence on sense of agency require a predictive model

Michiko Yoshie[1,2,*] and Patrick Haggard[3,*]

[1]Human Informatics Research Institute, National Institute of Advanced Industrial Science and Technology (AIST), Tsukuba, Ibaraki 305-8566, Japan

[2]Automotive Human Factors Research Center, AIST, Tsukuba, Ibaraki 305-8566, Japan

[3]Institute of Cognitive Neuroscience, University College London, London WC1N 3AR, United Kingdom

* e-mail: m.yoshie@aist.go.jp; p.haggard@ucl.ac.uk

List of Supplementary Information

Supplementary Results

Supplementary Tables

Supplementary Table S1. Results of two-way ANOVA for the composite binding

Supplementary Table S2. Results of two-way ANOVA for the sound shift

Supplementary Table S3. Results of two-way ANOVA for the action shift

Supplementary Table S4. Means (± SEM) of rank errors in the unpredictable condition

Supplementary Results

To help participants keep vigilance and to encourage their attention to the auditory stimuli, we asked participants to rank the frequency of the four sounds at the end of every sub-block. We used these data to investigate whether the unpredictable condition induced any biases in the perception of the frequency of negative and positive outcomes. We first calculated the difference between the actual rank orders of four sounds in each sub-block and those estimated by participants (*rank errors*) by subtracting the former from the latter (e.g., If the actual rank order of sounds A B C D is 3 1 4 2 and a participant's answer is 1 2 4 3, the rank errors are -2 1 0 1). A positive rank error indicated an underestimation of the frequency of the sound (sounds B and D in the above example), while a negative error indicated an overestimation of the frequency of the sound (sound A in the above example). For each sub-block of the unpredictable condition, we computed the mean rank errors separately for negative and positive sounds, and examined whether either negative or positive sounds were perceived to have appeared more frequently than the other (Supplementary Table S4). There was no significant difference in rank errors between negative and positive sounds in any of the three task conditions ($P > 0.05$, paired t-test, two-tailed). This demonstrates that the perceived frequency of negative outcomes was not different from that of positive outcomes.

Supplementary Tables

Supplementary Table S1. Results of two-way ANOVA for the composite binding

Effect	$F(1, 35)$	P	partial η^2
Valence predictability (V)	0.555	0.461	0.016
Outcome valence (O)	7.142	0.011	0.169
V × O	8.498	0.006	0.195

Supplementary Table S2. Results of two-way ANOVA for the sound shift

Effect	$F(1, 35)$	P	partial η^2
Valence predictability (V)	0.261	0.613	0.007
Outcome valence (O)	7.090	0.012	0.168
V × O	10.315	0.003	0.228

Supplementary Table S3. Results of two-way ANOVA for the action shift

Effect	$F(1, 35)$	P	partial η^2
Valence predictability (V)	0.420	0.521	0.012
Outcome valence (O)	0.504	0.482	0.014
V × O	0.008	0.931	0.000

Supplementary Table S4. Means (± SEM) of rank errors in the unpredictable condition

Task condition	Negative sounds	Positive sounds	*t*(35)	*P*
Agency action	0.0139 (0.0434)	-0.0139 (0.0434)	0.320	0.751
Agency sound	0.0035 (0.0371)	-0.0035 (0.0371)	0.094	0.926
Baseline sound	0.0035 (0.0378)	-0.0035 (0.0378)	0.092	0.927
